# Mesenteric Abscess Secondary to Panniculitis: A Rare Case Report

**DOI:** 10.7759/cureus.102514

**Published:** 2026-01-28

**Authors:** Pooja Babu lakshmanan, Madan Sundar, Ajay Gokul, Magesh Chandran, Kuberan Krishnan

**Affiliations:** 1 General Surgery, Sree Balaji Medical College and Hospital, Chennai, IND

**Keywords:** jejunal resection, laparotomy, mesenteric abscess, mesenteric panniculitis, panniculitis

## Abstract

Mesenteric panniculitis is an uncommon, chronic inflammatory disorder involving the adipose tissue of the mesentery. It can present with non-specific abdominal pain, fever, or gastrointestinal symptoms, and may occasionally progress to abscess formation. We report a case of a middle-aged patient who presented with intermittent left lower abdominal pain for five days, associated with fever and loose stools. Clinical examination revealed abdominal distension and tenderness in the right iliac fossa and umbilical regions. Imaging suggested inflammatory changes in the jejunal mesentery. Exploratory laparotomy revealed panniculitis-like changes in a jejunal loop 20 cm from the duodenojejunal (DJ) flexure and jejunal adhesions with a mesenteric abscess segment 25 cm from the DJ flexure. Resection of the affected segment was performed with an end-to-end jejunal anastomosis. Postoperative recovery was uneventful. Mesenteric panniculitis with abscess formation is rare and poses a diagnostic challenge. Early surgical intervention in symptomatic cases leads to favorable outcomes.

## Introduction

Mesenteric panniculitis (MP) is a rare, benign, non-neoplastic inflammatory disorder affecting the adipose tissue of the small-bowel mesentery [1]. First described by Jura in 1924, it represents a spectrum ranging from mesenteric lipodystrophy to retractile mesenteritis [2]. Although the terminology has varied historically, MP is now regarded as a single disease entity with variable inflammatory and fibrotic components [3,4]. The reported prevalence ranges from 0.2% to 7.8% on abdominal CT imaging, with most cases occurring in the fifth to seventh decades [5,6]. Epidemiologic data suggest a slight male predominance, although population-based variation may reflect geographic and reporting bias [7].

The etiology of MP remains unclear, with proposed associations including prior abdominal surgery, trauma, ischemia, autoimmune mechanisms, infection, metabolic dysfunction, and malignancy [8-10]. Most patients are asymptomatic, but symptomatic cases may present with abdominal pain, fever, altered bowel habits, or weight loss. Rare complications include bowel obstruction, ischemia, perforation, and abscess formation [11]. We report a rare case of jejunal mesenteric abscess secondary to panniculitis presenting as an acute abdomen.

## Case presentation

A 42-year-old male, a non-smoker and occasional alcoholic (consuming approximately 250 mL of alcohol twice a week), presented to the surgical outpatient department with complaints of left-sided lower abdominal pain for the past five days. The pain was insidious in onset, intermittent in nature, and aggravated on food intake, with no specific relieving factors. The patient also reported low-grade fever for four days, not associated with chills or rigors, and loose stools for three days, approximately three episodes per day, watery in consistency, and not mixed with mucus or blood. There was no history of vomiting, urinary complaints, previous abdominal surgery, weight loss, or tuberculosis.

On general examination, the patient was alert and oriented, afebrile (97.4°F), with a pulse rate of 92 beats/min, blood pressure of 140/80 mmHg, and oxygen saturation of 98% on room air. The abdominal circumference measured 96 cm. The patient was mildly dehydrated but hemodynamically stable. There was no pallor, icterus, cyanosis, clubbing, lymphadenopathy, or pedal edema. Systemic examination was unremarkable except for abdominal findings.

On abdominal examination, the abdomen was mildly distended with a central and everted umbilicus, and there were no visible peristaltic movements, scars, or pulsations. On palpation, there was localized tenderness in the right iliac fossa and umbilical regions, while the flanks were full but compressible. There was no guarding, rigidity, palpable mass, or organomegaly. Percussion revealed a resonant note throughout the abdomen, and bowel sounds were present and normal on auscultation.

Routine laboratory investigations showed mild leukocytosis with a total leukocyte count (TLC) of 12,300/mm³ (reference: 4,000-11,000/mm³), neutrophilic predominance at 82% (reference: 40-70%), and elevated C-reactive protein (CRP) of 26 mg/L (reference: <5 mg/L). Liver and renal function tests were within normal limits.

**Figure 1:**
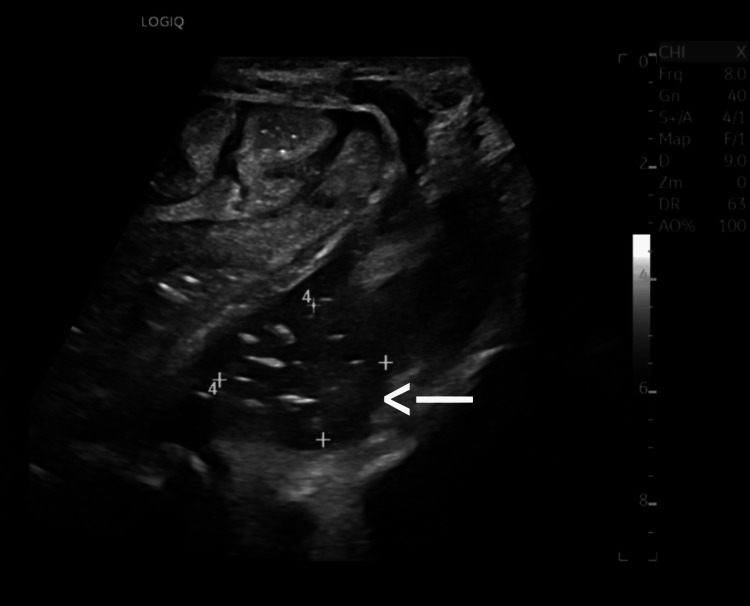
Ultrasonographic appearance of localized inflammatory changes in the proximal jejunal mesentery suggestive of early abscess formation (white arrow).

Ultrasound of the abdomen revealed a localized, echogenic, ill-defined area in the proximal jejunal mesentery, suggestive of inflammatory change or early abscess formation (Figure 1).

**Figure 2:**
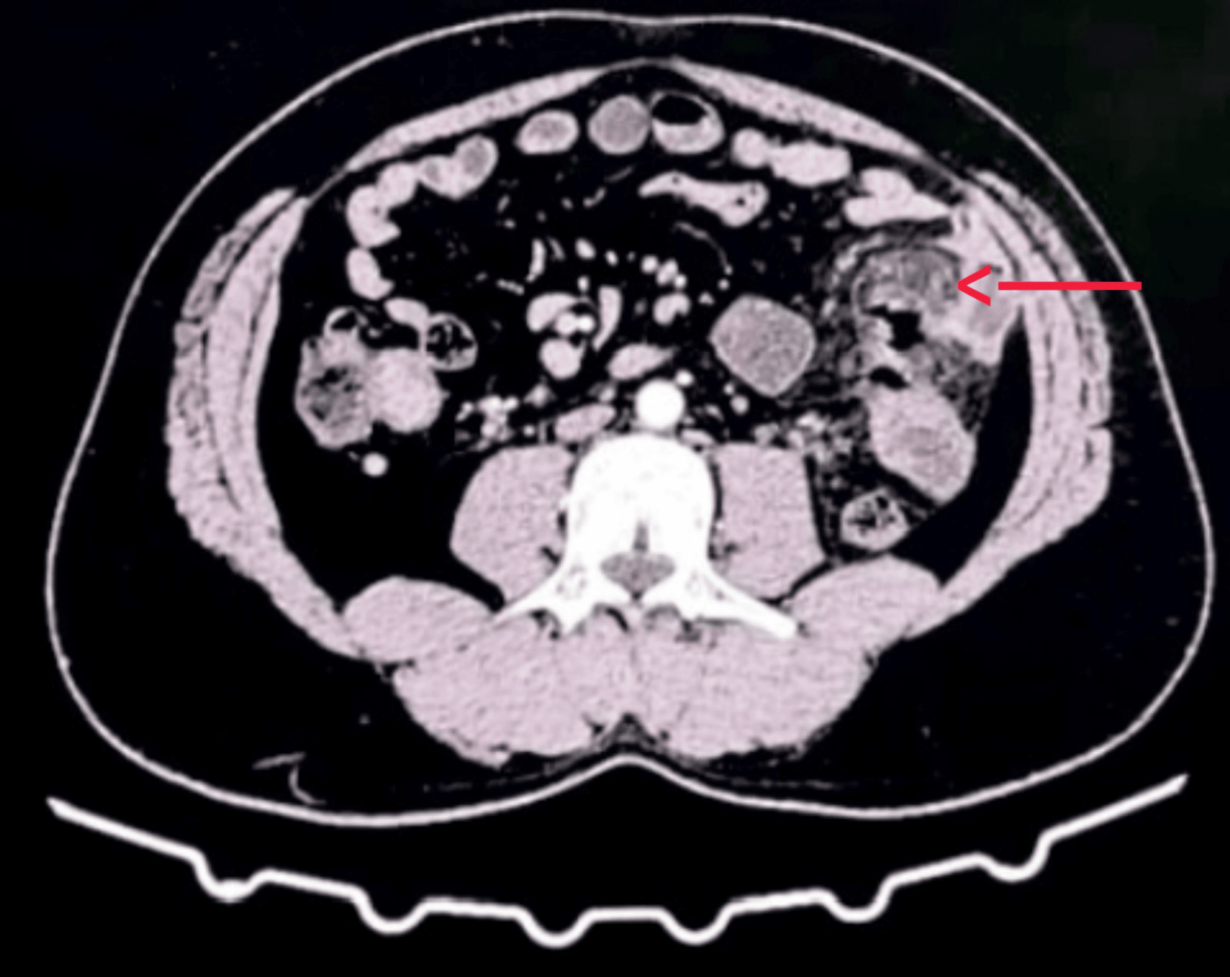
Contrast-enhanced CT abdomen showing increased mesenteric fat attenuation with mild soft-tissue stranding and a small adjacent fluid pocket near the jejunal loops (red arrow).

Contrast-enhanced CT (CECT) of the abdomen revealed an ill-defined area of increased fat attenuation within the proximal jejunal mesentery, associated with mild inflammatory stranding. Preservation of fat density surrounding the mesenteric vessels, consistent with the fat ring sign, was noted. No discrete enhancing soft-tissue mass or lymphadenopathy was identified, and a well-defined pseudocapsule was not appreciable. A localized hypodense collection with peripheral enhancement was seen within the inflamed mesentery, consistent with abscess formation (Figure 2). These imaging features were suggestive of MP with secondary abscess formation rather than a primary neoplastic or infectious process. The figure has been revised to include a digital arrow highlighting the area of interest, and the extraneous black background has been cropped as required.

In view of persistent localized tenderness and radiological findings, a decision for exploratory laparotomy was made.

**Figure 3:**
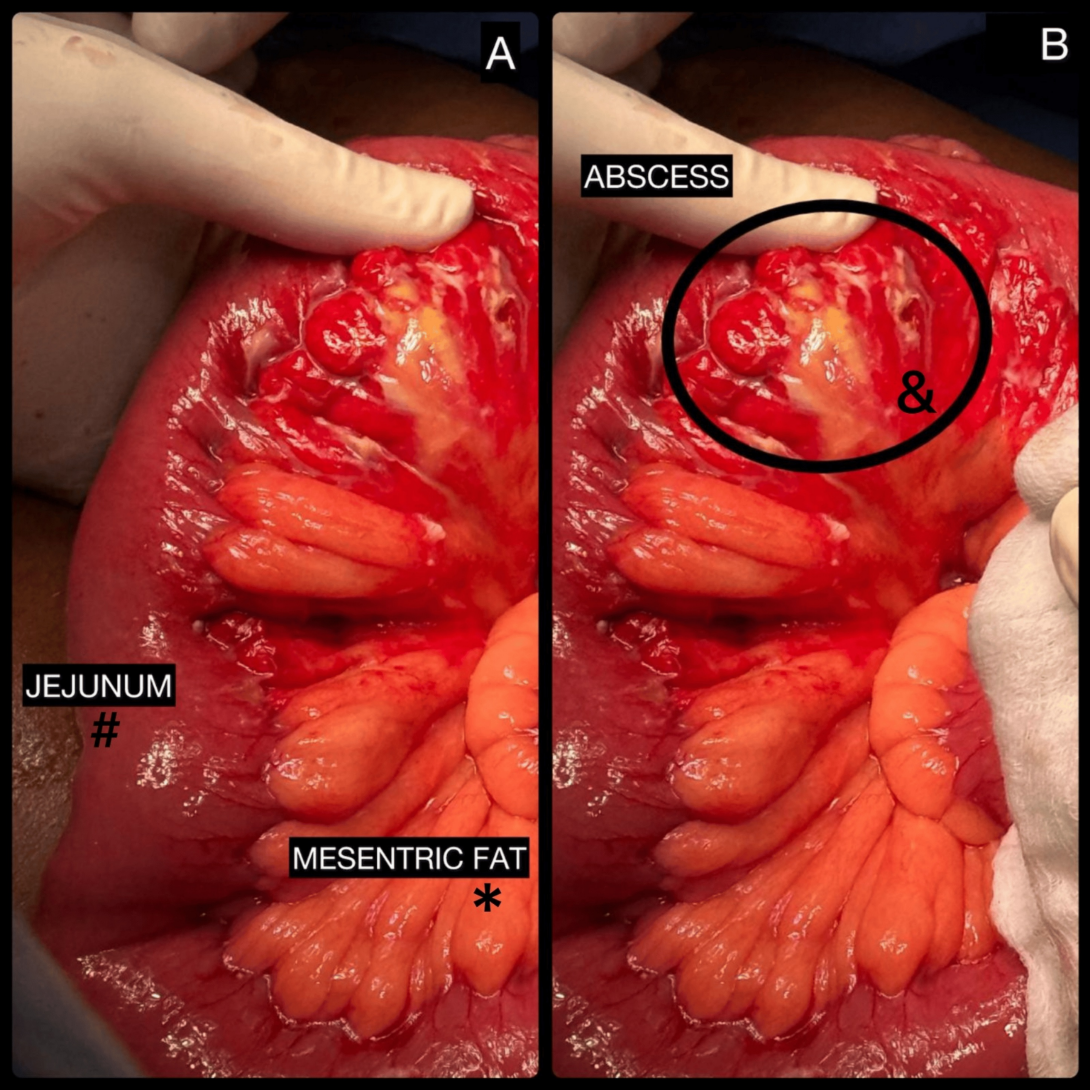
Intraoperative appearance of jejunal mesenteric abscess secondary to mesenteric panniculitis. (A) Inflamed jejunal loop with thickened, congested mesenteric fat consistent with panniculitis (#), with surrounding mesenteric fat involvement (*). (B) Localized mesenteric abscess adjacent to the jejunal loop (&) containing purulent material.

Under epidural anesthesia and strict aseptic precautions, the patient was placed in the supine position, and a midline laparotomy incision was made. The abdomen was opened in layers. Panniculitis-like changes were observed in a jejunal loop approximately 20 cm distal to the duodenojejunal (DJ) flexure, characterized by thickened, inflamed mesenteric fat with patchy areas of necrosis. Jejunal adhesions with a mesenteric abscess involving a segment 25 cm distal to the DJ flexure were identified (Figures 3A-3B). The abscess cavity contained approximately 20-25 mL of thick purulent material, which was drained and sent for bacterial culture and sensitivity. The remaining segments of the jejunum, ileum, and large bowel were normal. The liver, gallbladder, and other solid organs were grossly unremarkable.

**Figure 4:**
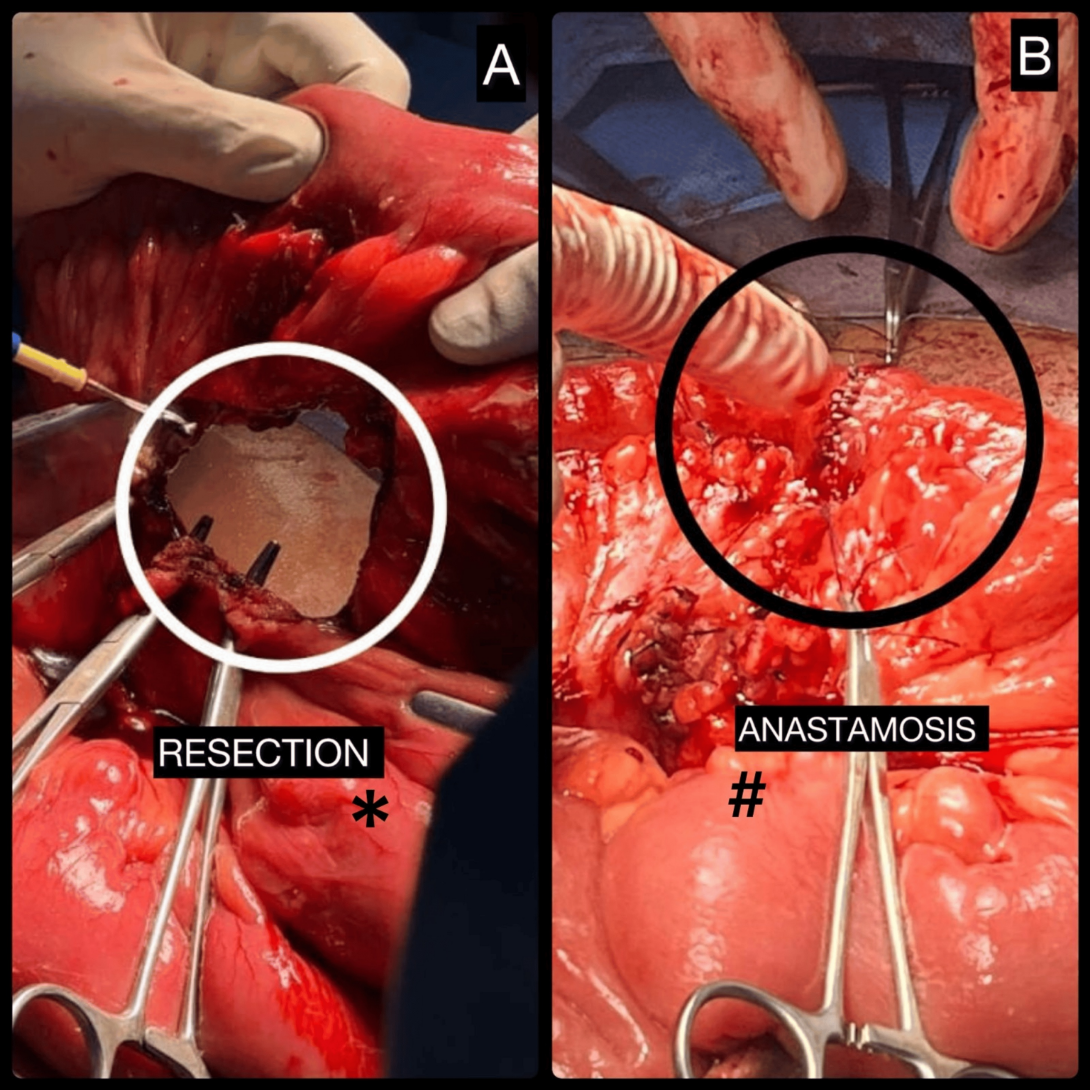
Segmental jejunal resection and primary jejunojejunal anastomosis. (A) Segmental resection of the affected jejunal segment along with the involved mesentery (*). (B) Completed hand-sewn, two-layer, end-to-end jejunojejunal anastomosis (#), demonstrating restoration of bowel continuity.

A segmental resection of the diseased jejunal segment with the involved mesentery was performed (Figure 4A), followed by a hand-sewn, two-layer, end-to-end jejunojejunal anastomosis (Figure 4B) using 3-0 Vicryl for the inner layer and 3-0 silk for the outer seromuscular layer.

A thorough peritoneal lavage was performed with warm normal saline. Two drains were placed-one near the anastomotic site and another in the pelvic cavity. Hemostasis was secured. The abdomen was closed in layers using loop Ethilon for the sheath and Ethilon interrupted sutures for the skin. A sterile compression dressing was applied.

The postoperative period was uneventful. The patient was managed with intravenous antibiotics (ceftriaxone and metronidazole), analgesics, and parenteral fluids. Bowel sounds returned on postoperative day 2, after which oral fluids were initiated and gradually advanced to a soft diet. Drain output reduced progressively and was serous by postoperative day 4. Drains were removed on day 5, and skin sutures were removed on day 10. The patient was discharged in stable condition.

Histopathological examination revealed fibrofatty mesenteric tissue with extensive fat necrosis, moderate lymphoplasmacytic inflammatory infiltration, and focal abscess formation. Areas of early fibrosis were present; however, dense sclerosing fibrosis was not observed. No granulomas, vasculitis, or malignant cells were identified. These findings were consistent with MP in the inflammatory stage, complicated by secondary abscess formation.

At three-month follow-up, the patient remained asymptomatic, with no recurrence of abdominal pain, fever, or bowel disturbances. Follow-up ultrasonography demonstrated complete resolution of the mesenteric inflammation and absence of residual fluid collection, confirming a favorable postoperative outcome. There are no standardized guidelines for long-term surveillance in MP. In asymptomatic patients with complete clinical and radiological resolution, routine imaging follow-up is generally not required, and clinical monitoring is considered sufficient. Recurrence is uncommon, particularly after resolution of the inflammatory process; however, repeat imaging may be warranted if symptoms recur or new abdominal complaints develop.

## Discussion

MP is a complex clinicopathologic entity that presents significant diagnostic and therapeutic challenges due to its nonspecific clinical features and radiologic overlap with other inflammatory and neoplastic conditions. The disorder is characterized histologically by varying degrees of chronic inflammation, fat necrosis, and fibrosis within the mesenteric fat. The underlying mechanisms are thought to represent an aberrant inflammatory response triggered by trauma, infection, autoimmune disease, or ischemia [8,9].

The diagnosis of MP is often incidental and made through abdominal CT, which remains the imaging modality of choice. Typical findings include a well-defined fatty mass at the root of the mesentery, increased fat attenuation (“misty mesentery”), small lymph nodes (<10 mm), and a hypodense halo (“fat ring sign”) surrounding mesenteric vessels and nodes [12]. The presence of a hyperdense pseudocapsule may also be noted. However, these findings are not pathognomonic and can mimic conditions such as lymphoma, carcinoid tumor, peritoneal carcinomatosis, desmoid tumor, or mesenteric tuberculosis. Therefore, differentiation from malignancy often requires histopathological confirmation, particularly in cases with nodules larger than 10 mm or atypical distribution.

Because MP can present radiologically and clinically as a soft-tissue mesenteric mass, the differential diagnosis is broad and includes both benign and malignant conditions. Table 1 summarizes key differential diagnoses for MP.

**Table 1:** Differential diagnosis of mesenteric abscess.

Category	Differential Diagnosis	Distinguishing Features
Infectious	Tuberculosis, histoplasmosis, actinomycosis	Granulomatous inflammation, positive culture/PCR
Neoplastic	Lymphoma, carcinoid tumor, desmoid tumor, sarcoma, metastases	Larger soft-tissue nodules, invasion, lymphadenopathy, systemic symptoms
Inflammatory/autoimmune	Retroperitoneal fibrosis, Crohn’s disease, sarcoidosis	Associated systemic autoimmune features
Miscellaneous	Post-surgical changes, ischemic fat necrosis, chronic abscess	History of surgery or infection, localized findings

Given this wide spectrum, the definitive diagnosis of MP often requires histopathological evaluation, which typically reveals a combination of fat necrosis, chronic inflammatory infiltrate (lymphocytes, plasma cells, histiocytes), and fibrosis. In the present case, infectious etiologies such as tuberculosis, histoplasmosis, and actinomycosis were considered but deemed unlikely due to the absence of granulomatous inflammation on histopathology, lack of systemic symptoms, and negative intraoperative and postoperative cultures. Neoplastic causes, including lymphoma, carcinoid tumor, desmoid tumor, sarcoma, and metastatic disease, were excluded based on contrast-enhanced CT findings that showed no discrete enhancing soft-tissue mass, invasive features, or significant lymphadenopathy, along with the absence of malignant cells on histological examination. Inflammatory and autoimmune conditions such as retroperitoneal fibrosis, Crohn’s disease, and sarcoidosis were ruled out due to the lack of systemic autoimmune manifestations, bowel wall involvement, or granulomas. Miscellaneous causes, including post-surgical changes, ischemic fat necrosis, and chronic abscess, were also considered unlikely given the absence of prior abdominal surgery or infection and the histopathological features consistent with MP. Collectively, these findings supported the final diagnosis of MP complicated by secondary abscess formation.

MP is most often a benign and self-limiting inflammatory condition. Progression to abscess formation is exceedingly rare, with only a few cases reported in the literature. In contrast to the usual indolent course managed conservatively, abscess formation represents a significant complication that may necessitate surgical intervention. The present case highlights this uncommon progression and underscores the importance of considering secondary abscess formation in patients with MP who present with acute abdominal symptoms or fail to respond to conservative management. In such cases, the clinical presentation may mimic acute appendicitis, perforated diverticulitis, or intra-abdominal abscess, making preoperative diagnosis difficult. In our case, the patient presented with fever, pain, and tenderness - a constellation suggestive of intra-abdominal sepsis - but the underlying cause (panniculitis with mesenteric abscess) could only be confirmed intraoperatively and histologically.

The management of MP depends on the clinical presentation. Asymptomatic or incidentally discovered MP requires no treatment and can be monitored. Symptomatic but uncomplicated cases may respond to conservative therapy, including corticosteroids, tamoxifen, colchicine, or immunomodulators such as azathioprine and thalidomide [13,14]. Infective or complicated forms (with abscess, obstruction, or perforation) necessitate surgical intervention, as in our case.

Surgical principles include resection of necrotic or abscess-bearing mesenteric tissue, drainage, and restoration of intestinal continuity via primary anastomosis if viable margins are available. Complete excision of the entire mesenteric lesion is rarely possible due to the involvement of vital vascular structures. Postoperatively, most patients recover uneventfully, though recurrence has been reported in fibrotic variants.

The prognosis of MP is generally benign, with most lesions remaining stable or regressing spontaneously. Only a small fraction progress to fibrosis or cause chronic symptoms. However, because MP has been associated with malignancy in up to 20-40% of cases in some series, long-term follow-up and periodic imaging are recommended to monitor disease course and to exclude underlying neoplasia [15].

This case emphasizes that MP should be considered among the differential diagnoses of mesenteric abscess or unexplained mesenteric masses, especially when the patient has a history of chronic inflammation or prior abdominal surgery. It also underlines the need for multidisciplinary evaluation - involving surgeons, radiologists, and pathologists - to avoid misdiagnosis and unnecessary radical resections.

## Conclusions

MP with abscess formation is an exceptionally rare manifestation of an already uncommon disorder. Because of its nonspecific presentation and imaging overlap with malignancy or infection, diagnosis is challenging. Early surgical exploration in complicated cases remains both diagnostic and therapeutic. Awareness of this entity and its diverse manifestations can help prevent misdiagnosis and guide timely management.
